# Cutaneous T-cell Lymphoma Progression: A Potential Dupilumab Pitfall

**DOI:** 10.7759/cureus.42959

**Published:** 2023-08-04

**Authors:** Hamza Malick, Allessia Wilson, Marshall Hall, Marshall Meier

**Affiliations:** 1 Medicine, Texas Agricultural and Mechanical College of Medicine, Dallas, USA; 2 Internal Medicine, Baylor University Medical Center, Dallas, USA

**Keywords:** cutaneous t-cell lymphoma, dupilumab, dupilumab dermatologic reaction, atopic eczema, cutaneous malignancy, atopic dermatitis

## Abstract

Dupilumab is a monoclonal antibody inhibiting key drivers in the inflammatory signaling cascade. It has been considered a saving grace for many patients suffering from severe atopic dermatitis and has quickly become a widespread treatment option for many inflammatory pathologies. However, recent reports have linked dupilumab with exacerbating symptoms and accelerated disease progression of cutaneous T-cell lymphoma (CTCL). This report presents the case of a 60-year-old African American female who was diagnosed with CTCL three months after her initial treatment with dupilumab for presumed atopic dermatitis. An appreciation of this unique relationship, as well as the morphological differences seen in patient presentation, is important for physicians to better guide timely diagnoses while working to uncover the true relationship between dupilumab and CTCL.

## Introduction

Dupilumab is a monoclonal antibody that antagonizes the alpha subunit of interleukin-4 (IL-4) and interleukin-13, thereby inhibiting key drivers in the inflammatory signaling cascade [1, 2]. It has emerged as a critical advancement in the treatment of severe atopic dermatitis and has gained widespread acceptance and notoriety in dermatological practice. However, there have been rare instances recently where the use of dupilumab has exacerbated symptoms and possibly contributed to disease progression in patients with underlying cutaneous T-cell lymphoma (CTCL). This report presents the case of an African American patient who experienced a worsening skin rash and hypopigmented lesions. Eventually, ten months after initiating dupilumab treatment for atopic dermatitis, she was diagnosed with cutaneous T-cell lymphoma.

## Case presentation

**Figure 1:**
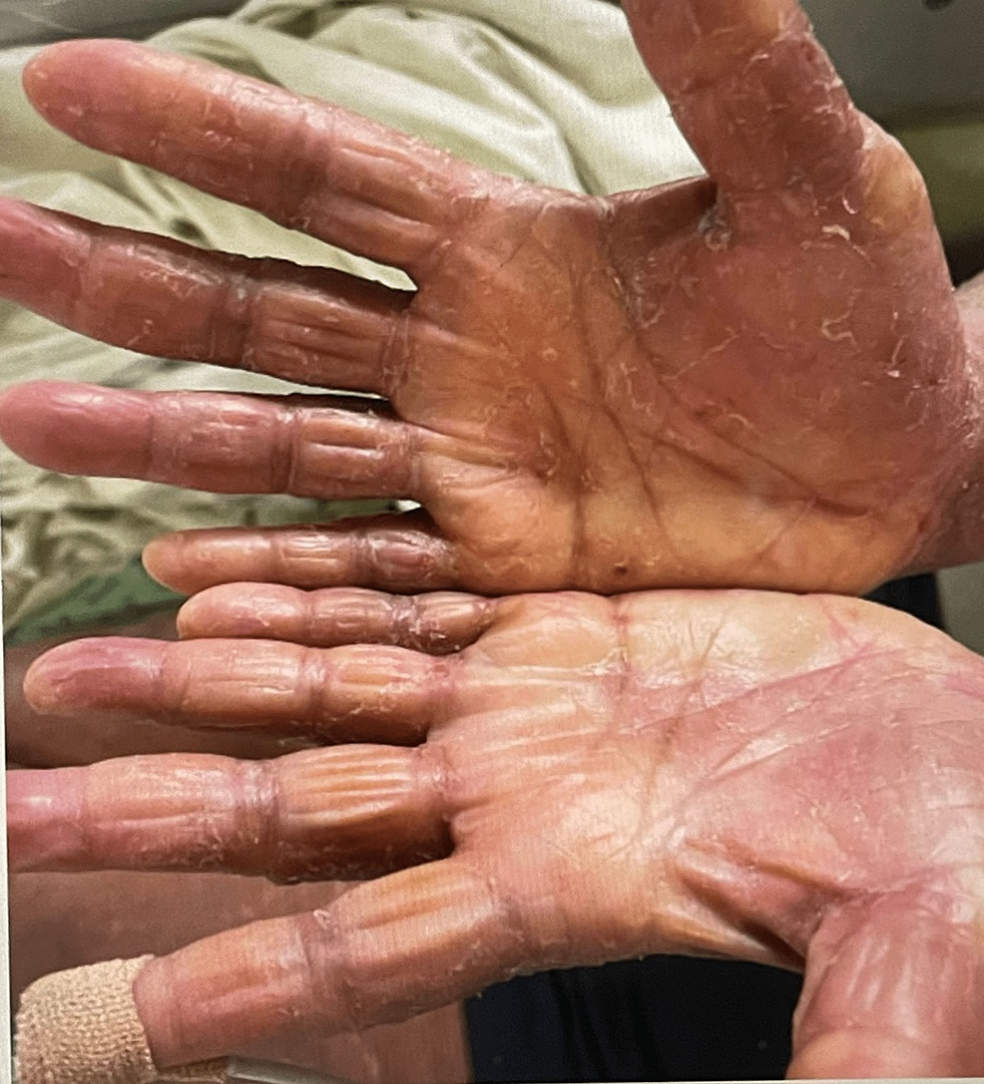
The rash evolved into a desquamating rash that spread to cover the entire body including acral and palmar surfaces

**Figure 2:**
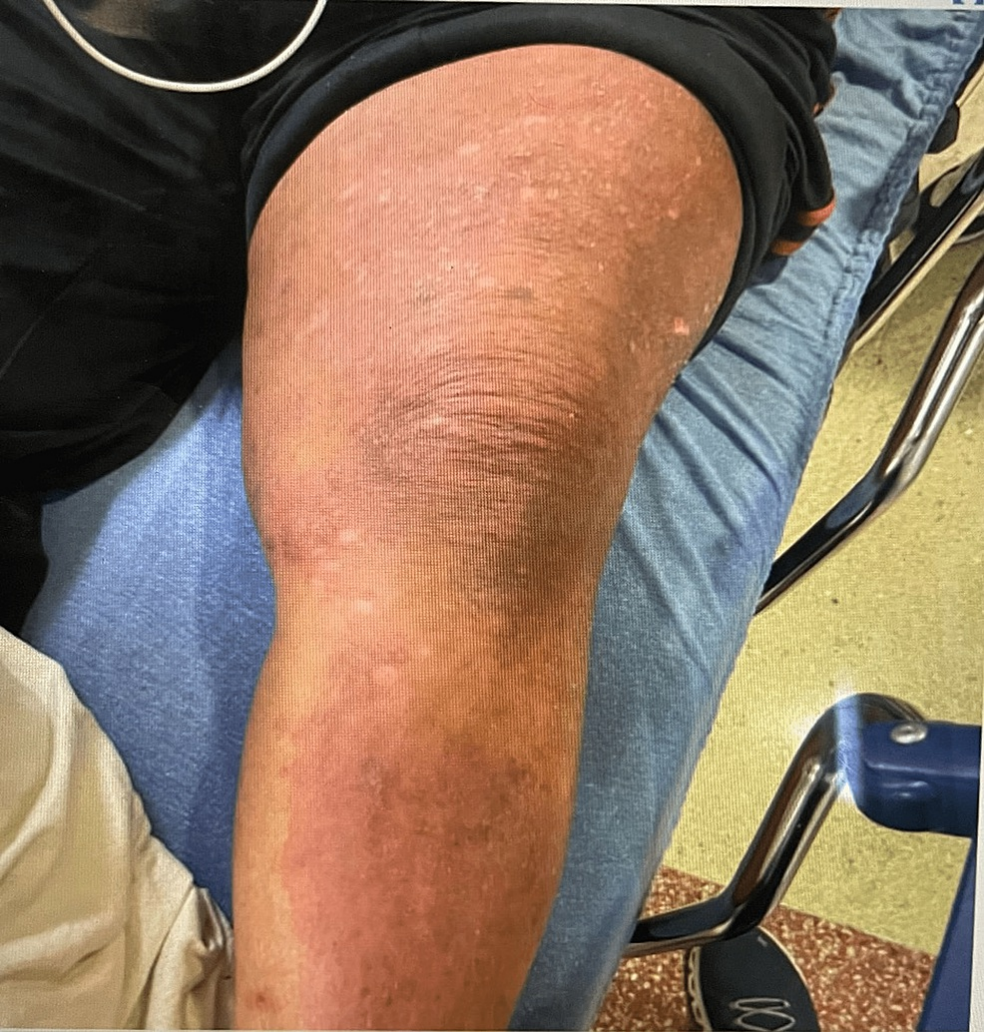
Patient presented with patches of erythematous desquamating skin and acute onset of hyper-pigmented plaques

A 60-year-old female with a past medical history of eczema, gastroesophageal reflux disease (GERD), fibromyalgia, type 2 diabetes mellitus, and osteoarthritis presented to the emergency department due to a worsening rash that had spread all over her body. The patient had a three-year history of mild-to-moderate eczema without any identifying triggers. She started dupilumab for her eczema three months prior to the current admission due to a persistent rash of the same intensity. However, since starting dupilumab therapy, the patient experienced a worsening rash with fluctuating periods of intensity. She previously visited the emergency department (ED) one month after starting dupilumab treatment due to worsening itch with spreading erythematous rash and was given a course of systemic steroids and antibiotics that showed partial resolution of symptoms. Her last injection of dupilumab was administered three weeks prior to her current presentation. Since the last dose, she had experienced a diffuse, desquamating rash without pustules, sloughing, or blistering. It initially appeared on her arms and thighs and gradually spread, now covering her entire body from scalp to toes, including areas on the lips and vulva (Figure 1). Her lower extremities further showed an acute onset of erythematous, hyperpigmented plaques (Figure 2). The rash caused severe itchiness and significant flaking of the skin, leading to distress and pain. The patient denied any recent changes in soaps, detergents, lotions, or medications.

She was hemodynamically stable and afebrile at presentation but exhibited significant atypical lymphocytosis. Additionally, new palpable lymphadenopathy was observed in the axillary and inguinal regions. Skin biopsies were performed, and the leukemia and lymphoma panel indicated 26.8% CD4+/CD7- cells. The patient was diagnosed with cutaneous T-cell lymphoma (CTCL) and started on a daily dose of 25mg of acitretin. She was also referred for extracorporeal photopheresis (ECP).

## Discussion

Dupilumab is a monoclonal antibody that antagonizes the interleukin-4 (IL-4) alpha subunit and interleukin-13, thereby inhibiting key drivers in the inflammatory signaling cascade [1, 2]. It has been hailed as a saving grace for many patients suffering from severe atopic dermatitis and has become a widely used treatment option. Initially, studies hypothesized that dupilumab's inhibition of IL-13 receptors would be an effective treatment for cutaneous T-cell lymphoma (CTCL) due to its dependence on IL-13 signaling for malignant cell proliferation [3]. However, recent reports have shown that dupilumab's modulation of these specific cytokines may actually accelerate tumor progression in patients with underlying CTCL [3, 4].

It is important to note that other documented cases have also reported a paradoxical worsening of skin scaling, lymphadenopathy, severe pruritis, and extension of rash borders one to two months after the initial administration of dupilumab [3,5]. The patient described in this case additionally presented with an acute onset of hyperpigmented plaques, which aligns with the varied morphologies of CTCL in African American patients [6,7]. The discernible erythematous plaques pathognomonic for CTCL in light-skinned individuals often contrast with the varying pigmentations with little erythema presented in darker-colored skin [7]. These variations in clinical presentation, along with the atypical manifestation of a desquamating rash, can further complicate a diagnosis. The connection between dupilumab use and CTCL remains uncertain, but current findings indicate a possible association with the rapid progression of undiagnosed CTCL [4,8]. Further research is needed to understand the precise relationship and whether dupilumab might trigger CTCL. Therefore, it is advisable to conduct thorough screening for risk factors and underlying CTCL in patients before initiating dupilumab treatment. Patients with hypopigmented lesions may be examined using Wood's light to delineate other causes of cutaneous hypopigmentation, including vitiligo, post-inflammatory hypopigmentation, dermatophyte infections, and melasma [9].

Adult-onset atopic dermatitis affects approximately 1%-3% of the general population. Its diverse symptoms and stubborn nature make it challenging to diagnose, often necessitating additional diagnostic studies, such as a biopsy of the affected skin [10]. Since the average time to diagnose cutaneous T-cell lymphoma (CTCL) after initiating dupilumab treatment is 7.8 months, and late-stage diagnoses significantly reduce five-year survival rates, it is crucial to recommend consistent follow-up and early skin biopsies for patients with adult-onset atopic dermatitis [8]. It may also be necessary to caution early intervention in patients without noted improvement in eczematous symptoms after dupilumab therapy in these patients, warranting updates to guidelines in medication management. Physicians should be knowledgeable about morphological differences in African American patients and be vigilant of the possible connection to this medication. An understanding of these important relationships will help guide appropriate follow-up care for patients.

## Conclusions

The approval of dupilumab for the treatment of moderate-to-severe atopic dermatitis has led to a significant expansion of its usage and exploration in various dermatological disorders. Consequently, it is crucial to provide medical professionals with an understanding of a potentially detrimental pitfall of dupilumab. This becomes increasingly important as dupilumab becomes a widely adopted treatment option for numerous inflammatory dermatological conditions, including atopic dermatitis. Furthermore, we aim to shed light on the variances in patient presentation in the skin of color patients with CTCL. The heightened morbidity and delayed treatment observed in such patients should prompt clinicians to recognize and educate themselves about morphological differences. Further studies are needed to evaluate this possible relationship and uncover the cellular mechanisms behind CTCL and dupilumab.
